# Expression analysis of genes including *Zfhx4* in mice and zebrafish reveals a temporospatial conserved molecular basis underlying craniofacial development

**DOI:** 10.1002/dvdy.740

**Published:** 2024-09-25

**Authors:** Shujie Liu, Lin Xu, Makoto Kashima, Rika Narumi, Yoshifumi Takahata, Eriko Nakamura, Hirotoshi Shibuya, Masaru Tamura, Yuki Shida, Toshihiro Inubushi, Yuko Nukada, Masaaki Miyazawa, Kenji Hata, Riko Nishimura, Takashi Yamashiro, Junichi Tasaki, Hiroshi Kurosaka

**Affiliations:** ^1^ R&D, Safety Science Research, Kao Corporation Kawasaki Japan; ^2^ Department of Orthodontics and Dentofacial Orthopedics Osaka University Graduate School of Dentistry Suita Japan; ^3^ Department of Chemistry and Biological Science College of Science and Engineering, Aoyama Gakuin University Sagamihara Japan; ^4^ Department of Biomolecular Science, Faculty of Science Toho University Funabashi Japan; ^5^ Department of Molecular and Cellular Biochemistry Osaka University Graduate School of Dentistry Suita Japan; ^6^ Mouse Phenotype Analysis Division, RIKEN BioResource Research Center Tsukuba Japan; ^7^ R&D, Safety Science Research, Kao Corporation Tochigi Japan

**Keywords:** cranial neural crest cells, disease models, loss‐of‐function analysis, palatogenesis, stage‐matched gene expression analysis, temporal expression patterns

## Abstract

**Background:**

Embryonic craniofacial development involves several cellular and molecular events that are evolutionarily conserved among vertebrates. Vertebrate models such as mice and zebrafish have been used to investigate the molecular and cellular etiologies underlying human craniofacial disorders, including orofacial clefts. However, the molecular mechanisms underlying embryonic development in these two species are unknown. Therefore, elucidating the shared mechanisms of craniofacial development between disease models is crucial to understanding the underlying mechanisms of phenotypes in individual species.

**Results:**

We selected mice and zebrafish as model organisms to compare various events during embryonic craniofacial development. We identified genes (*Sox9*, *Zfhx3* and *4*, *Cjun*, and *Six1*) exhibiting similar temporal expression patterns between these species through comprehensive and stage‐matched gene expression analyses. Expression analysis revealed similar gene expression in hypothetically corresponding tissues, such as the mice palate and zebrafish ethmoid plate. Furthermore, loss‐of‐function analysis of *Zfhx4/zfhx4*, a causative gene of human craniofacial anomalies including orofacial cleft, in both species resulted in deformed skeletal elements such as the palatine and ethmoid plate in mice and zebrafish, respectively.

**Conclusions:**

These results demonstrate that these disease models share common molecular mechanisms, highlighting their usefulness in modeling craniofacial defects in humans.

## Key Findings

- We identified genes (Sox9, Zfhx3 and 4, Cjun, and Six1) that exhibit similar temporospatial expression profiles during mice and zebrafish craniofacial development.
- The functional assay revealed craniofacial phenotypes, such as orofacial cleft, shared between mice and zebrafish by eliminating Zfhx4.
- zfhx4 in zebrafish plays critical roles in the development of cranial neural crest cells.

## 1 INTRODUCTION

Congenital anomalies exhibit different phenotypic features in various organs. Therefore, appropriate models are required to understand the cellular and molecular mechanisms underlying these diseases. Tissue‐specific cell lines that can recapitulate disease phenotypes have been used.^1^ The development of various organoids has enabled the investigation of the mechanisms underlying development and diseases at the specific level.^2^ However, we still require whole‐body disease models to investigate the etiology of systemic diseases that harbor congenital phenotypes in multiple organs.

Mice and zebrafish are the most frequently used disease models in modeling human diseases, such as congenital craniofacial defects, including orofacial clefts.^3^, ^4^ Understanding the mechanisms of embryonic facial process development is imperative to assess the phenotype of these two orofacial cleft models. At the onset of craniofacial development, cranial neural crest cells (CNCCs), a subpopulation of neural crest cells, are formed during neurulation. CNCCs migrate via the frontonasal and maxillary pathways, forming frontonasal and maxillary processes. The primary and secondary palates in humans are derived from the frontonasal and maxillary processes, respectively. Secondary palatal shelves develop and undergo vertical growth, elevation, horizontal growth, and fusion to complete the process. However, defects in these processes can result in orofacial clefts. Mouse palatogenesis mostly recapitulates these processes and is, thus, the most frequently used disease model for orofacial clefts.^5^ However, mice with human orofacial cleft gene knockout may not invariably replicate this phenotype.^6^ Furthermore, zebrafish have been used to explore orofacial cleft etiology (Liu et al., 2020).^7^, ^8^, ^9^, ^10^, ^11^, ^12^ In zebrafish, the anterior neurocranium (ethmoid plate) is functionally equivalent to the mammalian palate. This zebrafish “palate” originates from CNCCs. CNCCs separately migrate along the frontonasal and maxillary pathways once the cranial neural crest is formed. The anteriormost CNCCs migrate via the frontonasal pathway, whereas other CNCCs migrate via the maxillary pathway into the first pharyngeal arch (PA1). This CNCC stream generates the frontonasal and maxillary process, eventually forming the zebrafish “palate.”^8^, ^11^, ^13^, ^14^, ^15^, ^16^

Zebrafish palatogenesis starts with the convergence of the median element derived from the frontonasal process and two cartilage rods derived from the maxillary process in the middle of the face. While zebrafish palatogenesis is morphologically distinct from mammals, several common molecular networks and CNCC behaviors exist between zebrafish and mammals.^5^, ^17^ Despite each model system exhibiting species‐specific characteristics, conserved cellular and molecular mechanisms in both models enable studying the detailed etiology of craniofacial anomalies, including orofacial clefts, in humans. Although both species are used to model congenital anomalies, some aspects of the molecular mechanisms underlying the dynamics of embryonic development in these two species remain unelucidated.

*Sox9* is an essential gene for early craniofacial development and skeletogenesis in mice and zebrafish. *Sox9* loss‐of‐function results in severe craniofacial defects, including facial clefts in both species.^18^, ^19^ In this study, we conducted a comparative transcriptomic analysis of the mouse and zebrafish embryos at the corresponding developmental stages from neural crest cell migration to palatogenesis. We selected *Sox9* to match the skeletogenesis stages in these species to establish a molecular reference for comparison. Notably, our RNA‐Seq analysis of the consecutive embryonic stages in both developing models showed that *Sox9* and *sox9b*, but not *sox9a*, contained similar trajectories of dose expression patterns between these species. We hypothesized that these genes could show similar expression dynamics to *Sox9* orthologs during the developmental stage to identify critical genes in craniofacial skeletogenesis.^20^ Therefore, we selected 86 genes exhibiting expression dynamics similar to those of *Sox9* in mice and zebrafish. Consequently, we isolated several genes in both species, including *Zfhx3*, *Zfhx4*, *Cjun*, and *Six1*, whose expression was detected in developing craniofacial structures, especially in the upper jaw area. We further investigated the *Zfhx4/zfhx4* function during craniofacial development using mouse and zebrafish models, focusing on CNCC behavior and palatogenesis. Although *ZFHX4* is a novel pathogenic gene associated with orofacial clefts in humans, its role in craniofacial development is largely unknown. Deformed palatine and sphenoid bones in mice and zebrafish, respectively, were observed. Furthermore, we imaged CNCC behavior (migration and PA1 formation) and observed impaired CNCC behavior.

## 2 RESULTS

### 2.1 Genes with similar expression trajectories between mice and zebrafish

**FIGURE 1:**
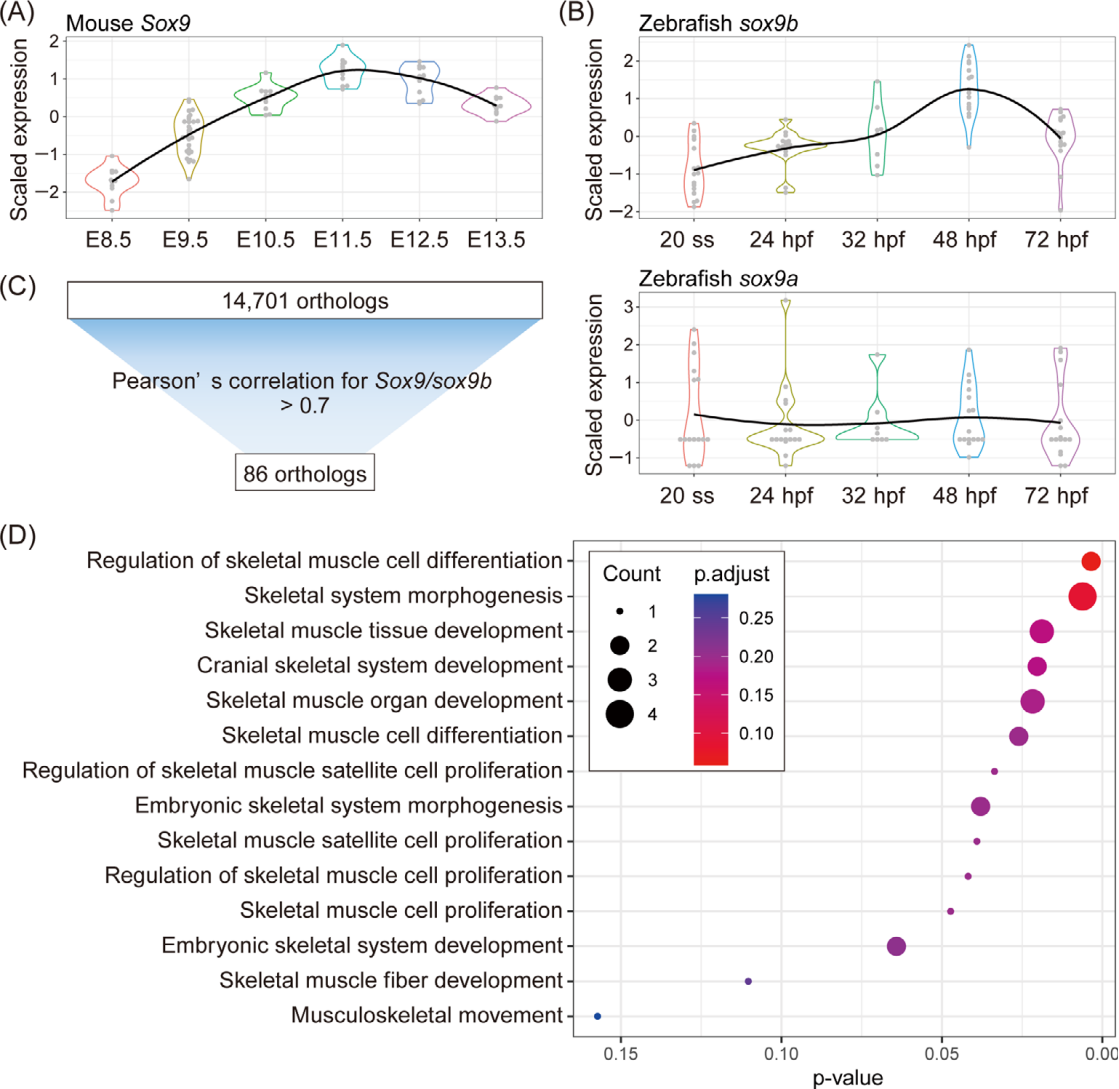
Screening of genes with similar trajectories between mice and zebrafish during craniofacial development. (A,B) Violin plots of scaled log2 normalized gene expression of *Sox9* orthologs in (A) mice and (B) zebrafish. (C) Criteria for identifying highly correlated orthologs with *Sox9* and *sox9b*. (D) Enrichment analysis results of the gene ontology terms, including “skeletal” for the 86 mouse orthologs, including *Sox9*.

We performed time‐course RNA‐Seq analysis to identify genes showing similar temporal and spatial expression dynamics during palatogenesis in mice and zebrafish. We obtained the whole body of mouse embryos spanning E8.5–E13.5 and zebrafish embryos from the 20‐somite stage (ss) at 72 h postfertilization (hpf) because of the wide cell distribution implicated in palatogenesis and the challenge of dissecting equivalent regions in early embryogenesis. We then compared the molecular profiles of the developmental stages in these two species, which exhibit analogous craniofacial developmental processes.^5^, ^21^ Consistent with the histological similarities, our RNA‐Seq results showed similar expression dynamics between *Sox9* in mice and *sox9b*, but not *sox9a*, in zebrafish (Figure 1A,B). These expression levels were relatively low at the initiation stage of developing CNCCs at E8.5 in mice and 20 ss in zebrafish. The expression levels gradually increased until E11.5 and 48 hpf in zebrafish, when the cartilage primordium developed in the craniofacial area in both species.^18^, ^19^ Considering the significance of *Sox9* in craniofacial development, we assumed that temporal similarity in gene expression could be linked to functional similarities with evolutionary conservation, as function loss leads to a cleft palate and craniofacial bone deformities.

For this study, we selected *Sox9* as a reference gene to orchestrate staging procedures and select other genes that could have a conserved function during craniofacial development. This choice is underpinned by its resemblance to the expression pattern throughout the neural crest development in both species.^18^, ^19^ We identified 86 out of 14,701 orthologs by calculating the Pearson's correlation coefficient of gene expression levels between *Sox9/sox9b* and each orthologous gene, with Pearson's correlation coefficient >0.7 (Figure 1C and Table S1). Gene ontology (GO) enrichment analysis of the 86 genes in mice showed enrichment in the genes related to “cranial skeletal system development” (Figure 1D and Table S2). Based on our experience and literature reviews, we selected genes from the 86 orthologs to investigate the molecular similarities in craniofacial development between both species (Table S1). Notably, Irf6, which is a gene that induces cleft palates in mice and humans, is sometimes expressed within the embryonic maxillary process of zebrafish and serves a similar role between all three species.^8^, ^17^ Nevertheless, our gene catalog encompassed factors such as *Zfhx3*, *Zfhx4*, *Cjun*, *and Six1* that are pivotal in craniofacial development in mice and humans. However, their temporospatial expression patterns and functional involvement in zebrafish craniofacial development remain unclear.^22^, ^23^, ^24^, ^25^

### 2.2 Spatiotemporal expression of selected genes exhibited craniofacial expression

**FIGURE 2:**
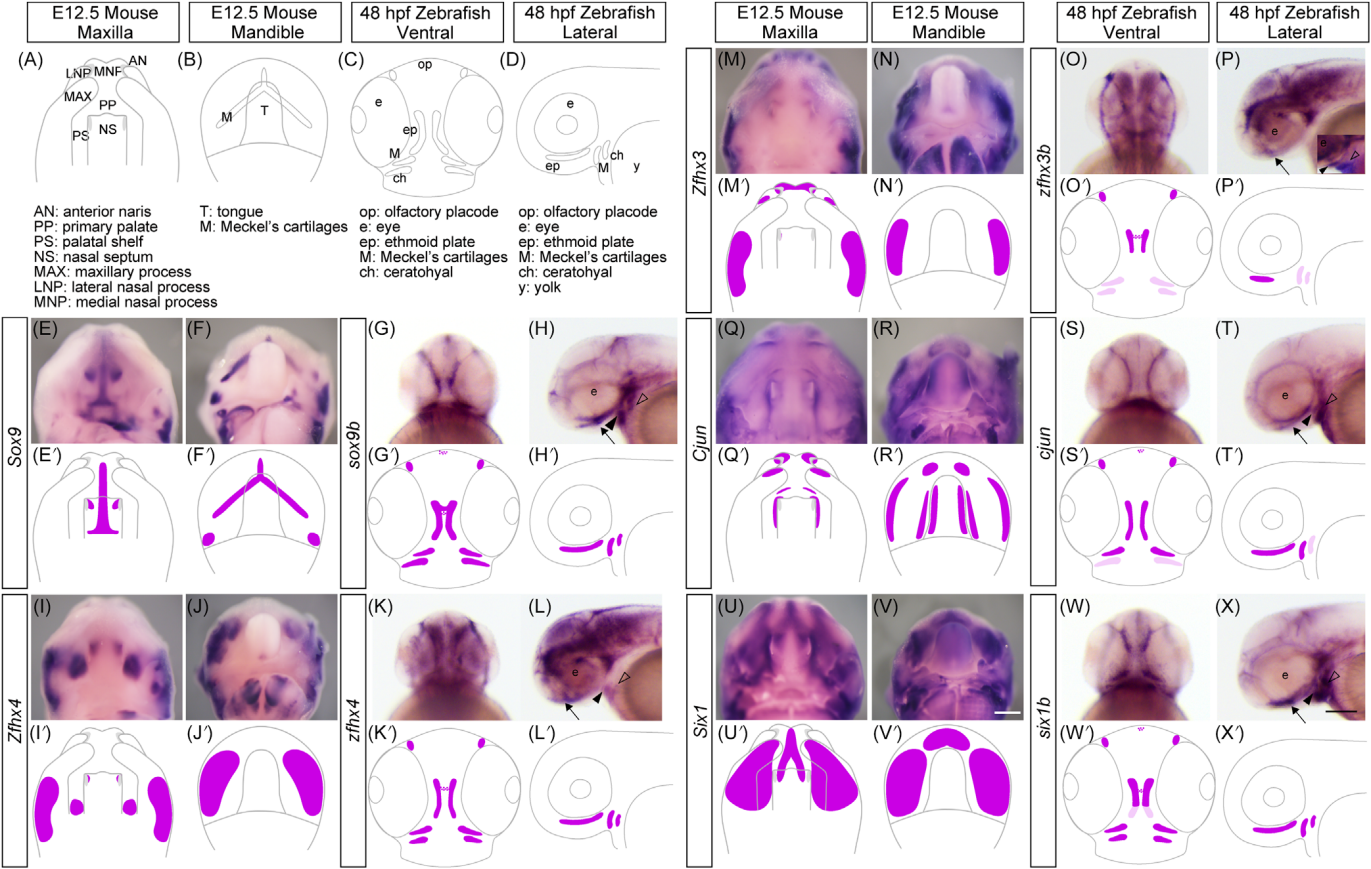
Comparing the expression pattern of the selected genes during craniofacial morphogenesis in mouse and zebrafish embryos. (A–D) Schematic illustrations of the craniofacial anatomy of the maxillary (A) and mandibular (B) regions of mice at E12.5 and zebrafish at 48 hours postfertilization (hpf) (C,D). (E–X) The expression pattern of *Sox9* (*sox9b*), *Zfhx4* (*zfhx4*), *Zfhx3* (*zfhx3b*), *Cjun* (*cjun*), and *Six1* (*six1b*) were examined using whole‐mount in situ hybridization. The samples are shown at the top, and the genes are shown on the left. (E′–X′) Schematic summary of the expression pattern of the selected genes. Black arrows, black arrowheads, and white arrowheads indicate Meckel's cartilages, ethmoid plate, and ceratohyal, respectively. Scale bars: 500 μm in V; 200 μm in X.

In situ hybridization of the selected genes was performed to detect temporospatial expression patterns in the developing heads of E12.5 mice and 48 hpf zebrafish with anatomical structures described (Figure 2A–D). In the E12.5 mouse maxilla, *Sox9* was expressed in the primary palate where cartilaginous tissue developed, such as in the nasal septum (Figure 2E,E′). Additionally, Meckel's cartilage development in the mandible indicated intense overall expression (Figure 2F,F′). At an equivalent developmental stage in zebrafish, *Sox9b* was expressed in the ethmoid plate, olfactory placode, and Meckel's cartilage (Figure 2G,G′,H,H′). *Zfhx4* was strongly expressed in the palatal shelf (Figure 2I,I′) and the proximal end of the mandible in mice (Figure 2J,J′). The ethmoid plate and mandible also exhibited strong *Zfhx4* expression in zebrafish, indicating the essential roles of *Zfhx4* in embryonic craniofacial structures in both species (Figure 2K,K′L,L′). Another *Zfhx* family gene, *Zfhx3*, showed a relatively restricted expression pattern around the ventrolateral edge of the anterior developing nasal septum with medial and lateral nasal processes in the mouse maxilla (Figure 2M,M′). The developing alveolar area exhibited strong expression in the mandible, resembling the *Zfhx4* profile (Figure 2N,N′). Additionally, the *zfhx3b* expression pattern resembled that of *zfhx4* in the zebrafish head, showing broad expression in the ethmoid plate and mandible (Figure 2O,O′,P,P′). In the mouse maxilla, *Cjun* was expressed at the palatal shelf and upper incisors of the primary palate (Figure 2Q,Q′). The mandibular incisors also exhibited strong expression at the anterior end of the mandible, together with the lateral edge of the tongue (Figure 2R,R′). Additionally, *Cjun* expression was relatively mild and ubiquitous throughout the zebrafish ethmoid plate and Meckel's cartilage (Figure 2S,S′,T,T′). *Six1* was detected throughout the primary palate and palate shelf in mice. Similar to the maxilla, a large area of the mandible was observed in mice (Figure 2U,U′,V,V′). Furthermore, *Six1b* expression in zebrafish resembled that of *Sox9b*, which was strongly expressed in the ethmoid plate and Meckel's cartilage (Figure 2W,W′,X,X′).

**FIGURE 3:**
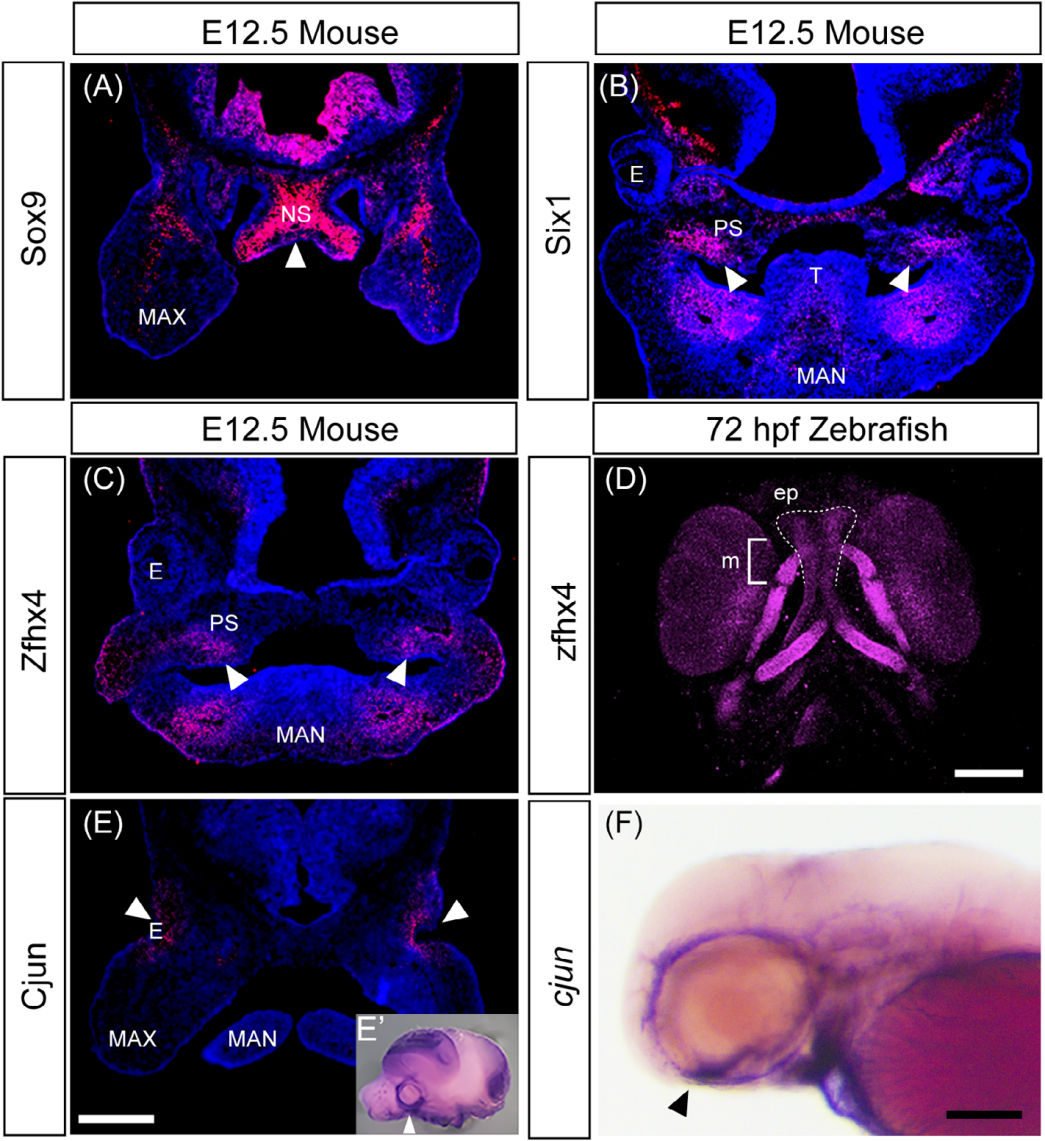
Protein localization in the developing head of mice and zebrafish. (A–D) Immunohistochemistry of selected transcription factors involved in chondrogenesis and osteogenesis using frontal sections of E12.5 mice head (A–C) and ventral view of 72 hours postfertilization (hpf) zebrafish larva (D). Samples are shown at the top, and proteins are shown on the left. White arrows indicate the protein detected in the nasal septum (A) and palate (B,C). (D) Immunofluorescence staining with anti‐*Zfhx4* antibody in zebrafish embryos at 72 hpf. The developing ethmoid plate (zebrafish palate, dashed line) and Meckel's cartilage (mandibular, blacked) were stained with the antibody. *Zfhx4* was also detected in the eye, ceratobranchial, ceratohyal, and hyosymplectic. (E,F) Similar expression domains of *Cjun* in both species were revealed using immunohistochemistry in the frontal section of E12.5 mice head, (E) *cjun* mRNA in the lateral view of E12.5 mice head, (E′) and the lateral view of 72 hpf zebrafish (F). Arrowheads indicate the expression surrounding the ocular region. E, eye; ep, ethmoid plate; MAN, mandibular; MAX, maxillary; m, Meckel's cartilage; NS, nasal septum; PS, palatal shelf; T: tongue. Scale bars: 500 μm in (E); 100 μm in (D,F).

Immunohistochemistry for specific factors was performed in mouse embryos to compare mRNA expression and protein localization during mouse development. SOX9 accumulated in the cartilaginous mesenchymal tissue of the nasal septum of mice (Figure 3A). SIX1 and ZFHX4 were present in the developing skeletal mesenchyme tissue in the secondary palate of mice, confirming the mRNA expression pattern (Figure 3B,C). Additionally, *zfhx4* expression was confirmed in zebrafish, which exhibited strong expression in craniofacial skeletal elements, such as the ethmoid plate (Figure 3D, dashed line). CJUN exhibited intense expression in the mesenchymal tissue around the developing eye in mice (Figure 3E,E′), which was also evident in in situ hybridization of zebrafish (Figure 3F, arrowhead). Unfortunately, the ZFHX4 antibody was the only antibody capable of detecting signals in zebrafish among the antibodies we used, and other protein localizations in zebrafish require further investigation.

### 2.3 Phenotypic similarity between zebrafish and mice for eliminating *Zfhx4/zfhx4*

**FIGURE 4:**
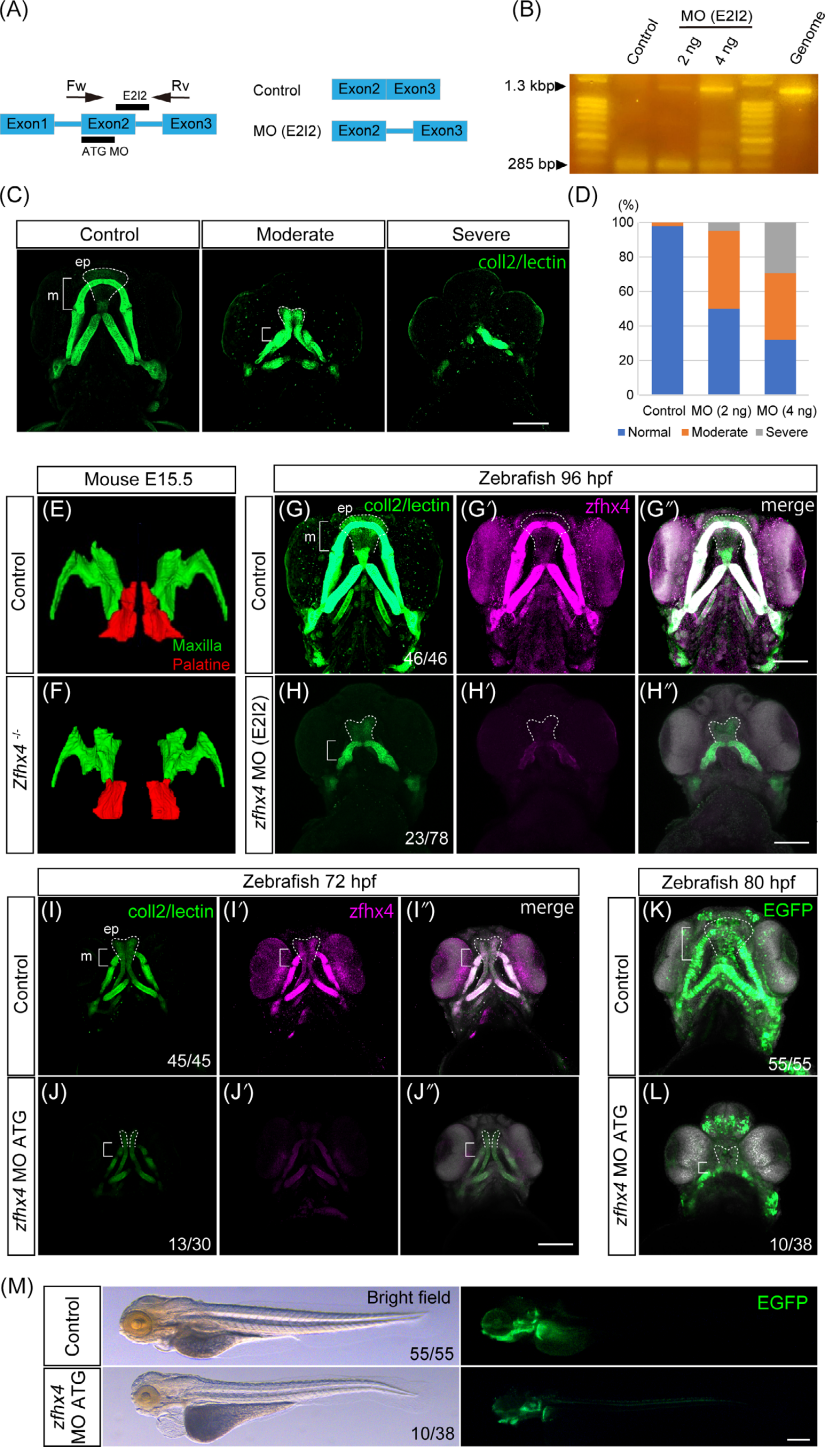
Morpholino (MO)‐based *zfhx4* function loss led to craniofacial malformation in zebrafish. (A) Pre‐mRNA of *zfhx4* and splicing MO design. Splicing MO (E2I2) was designed to bind to the exon 2 and intron 2 boundary. Presumptive *zfhx4* mRNA inhibited by the MO (E2I2) showed splice site skipping between exon 2 and intron 2. (B) Splice‐blocking efficiency confirmation of the MO (E2I2) using reverse transcription‐polymerase chain reaction (RT‐PCR). Total RNA was isolated from the control and *zfhx4* morphants at 1 day postfertilization. Forward and reverse primers were designed to target exons 2 and 3, respectively (A). PCR product length of the control was 285 bp, and the length of the splice‐inhibited products was 1305 bp. A single band was detected at the 285 bp length in the control. An unspliced product containing intron 2 was detected at the 1.3 kbp length in the *zfhx4* morphants. This band was observed in the same position as the genomic PCR product. (C,D) Typical *zfhx4* morphants exhibited moderate and severe craniofacial anomalies. (D). Frequency of the craniofacial anomalies in the *zfhx4* morphants. (Control: *N* = 46, MO [E2I2] of 2 ng/embryo: *N* = 62, MO [E2I2] of 4 ng/embryo: *N* = 28). Phenotypic severity was observed with MO (E2I2) in a dose‐dependent manner, corresponding to the RT‐PCR result in Figure 4B. (E,F′) Three‐dimensional reconstructed maxillary (green) and palatine bones (red) in E15.5 control and *Zfhx4* null embryos. Smaller maxillary and deformed palatine bones were observed in *Zfhx4* null mice compared with their littermate controls. (G–H″) *zfhx4* morphants displayed cleft palate and micrognathia (G,H). Effective functional *zfhx4* inhibition was confirmed by decreased *Zfhx4* expression via immunofluorescence staining (G–H″, Control: *N* = 46, MO [E2I2]: *N* = 78). ATG MO of *zfhx4* showed craniofacial defects similar to those of E212 MO (I–L). (M) Overall morphology of *zfhx4* morphant at 80 hpf. (Control: *N* = 45, ATG MO: *N* = 30 in I–J″). (Control: *N* = 55, ATG MO: *N* = 38 in K–M). ep, ethmoid plate; m, Meckel's cartilage. Scale bars: 100 μm.

*Zfhx4/zfhx4* elimination was performed in mice and zebrafish for functional assessment because of its indispensable involvement in palatogenesis and status as a genetic contributor to orofacial clefts in humans,^23^ A *zfhx4* splicing morpholino (MO; E2I2) was designed to bind to the border between exon 2 and intron 2 of *zfhx4* to inhibit splicing in zebrafish (Figure 4A). Efficient splicing inhibition of *zfhx4* by MO was confirmed using real‐time reverse transcription‐polymerase chain reaction (RT‐PCR) in the treated embryos (Figure 4A,B). The phenotype exhibited a dose‐dependent effect, with a further severe phenotype in the micrognathia and a cleft palate at higher MO doses (Figure 4C,D). *Zfhx4* null mice exhibited craniofacial defects, including a secondary cleft palate with 100% penetrance.^22^ Additionally, we analyzed the three‐dimensional reconstructed anatomy of the maxillary and palatine bones using micro‐computed tomography (CT). The maxillary and palatine bones exhibited morphological differences between the control and *Zfhx4* null mice (Figure 4E,F). The anterior portion of the palatine bone was missing in the E15.5 *Zfhx4* null mice (Figure 4E,F). Immunohistochemistry of MO‐treated zebrafish indicated reduced overall immunoreactivity of *zfhx4* in the skeletal elements (Figure 4G',H'). The skeletal morphology exhibited a shorter ethmoid plate and Meckel's cartilage, resulting in an overall craniofacial distortion and a phenotype similar to that in mice (Figure 4G–G'', H–H''). Additionally, ATG MO was designed to bind to the translational initiation site of *zfhx4*. The expression of *zfhx4* was severely decreased by ATG MO (Figure 4I',J'). Craniofacial malformations such as micrognathia and cleft palate were observed in the morphants (Figure 4I″,J″). These morphological defects were also observed at later stage (80 hpf), similar to those of phenotypes caused by E2I2 MO (Figure 4K,L). In addition to craniofacial defects, gross morphological defects such as short stature and cardiac defects were observed in the morphants (Figure 4M). These findings indicate that disrupting a common gene may distort the ethmoid plate in zebrafish and the palatal shelf in mice. This result motivated us to investigate whether *Zfhx4/zfhx4* shares potential relevance in contributing to neural crest development.

### 2.4 Lineage tracing of CNCCs involved in ethmoid plate development in zebrafish

**FIGURE 5:**
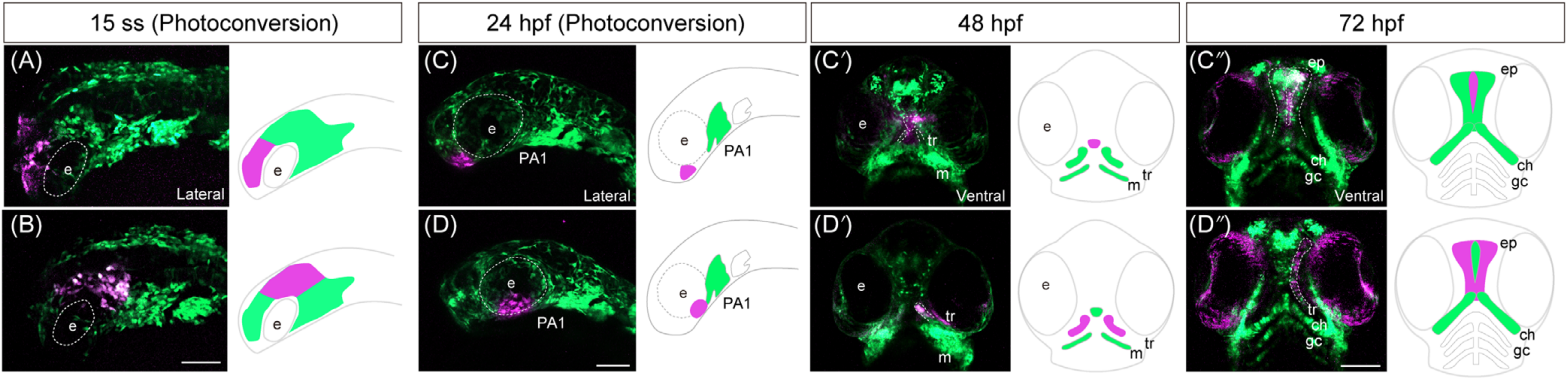
Lineage tracing of cranial neural crest cells (CNCCs) in *sox10:Dendra2*. (A,B) For time‐lapse imaging of the prospective frontonasal and anterior maxillary prominence, photoconversion of the prospective frontonasal prominence (A) and prospective anterior maxillary prominence (B) was performed in *sox10:Dendra2* transgenic zebrafish at 15 ss. As photoconvertible fluorescent protein Dendra2 is photoactivated by UV light (405 nm) from green to red, specific Dendra2‐expressing cells were labeled irreversibly and traced. The time‐lapse imaging was performed by 30 ss (Movies [Link], [Link], [Link]). (C–C″) To investigate the cell lineage of the prospective frontonasal prominence after 30 ss, photoconversion of the frontonasal prominence at 24 hpf (30 ss) were performed (C). The lineage was subsequently examined at 48 (C′) and 72 hpf (C″). The frontonasal prominence was labeled at 24 hpf (C) and differentiated into the medial part of the ethmoid plate at 48 hpf (C′) and 72 hpf (C″). (D–D″) To investigate the cell lineage of the prospective anterior maxillary prominence after 30 ss, photoconversion of the anterior maxillary prominence at 24 hpf (30 ss) were performed (D). The lineage was subsequently examined at 48 hpf (D′) and 72 hpf (D″). The anterior maxillary prominence was labeled at 24 hpf (D) and differentiated into a lateral part of the ethmoid plate (trabeculae) at 48 hpf (D′) and 72 hpf (D″). e, eye; ch, ceratohyal; ep, ethmoid plate; gc, gill cartilage; m, Meckel's cartilage; PA1, first pharyngeal arch. Scale bars: 100 μm.

Several studies reported that two distinct CNCC streams populate in the ethmoid plate.^9^, ^17^, ^29^ To elucidate the developmental dynamics of CNCCs during ethmoid plate development, lineage tracing of CNCCs was performed using *sox10:Dendra2*. The prospective frontonasal region at the 15 ss was labeled (Figure 5A) and traced chronologically by 30 ss (Movie S1). The labeled CNCC at 15 ss (Figure 5A magenta) migrated along the dorsal side of the eye and were destined for the frontonasal region at 30 ss (Movie S1). To examine lineage of the frontonasal region after 30 ss, the frontonasal region at 24 hpf (30 ss; Figure 5C) in *sox10:Dendra2* was photoconverted, enabling us to trace its destination at 48 and 72 hpf. The result showed that the photoconverted region (magenta) was differentiated and incorporated into the middle of the ethmoid plate at 48 hpf (Figure 5C′), where it persisted at 72 hpf (Figure 5C″). Thus, the majority of the prospective frontonasal region comprises the middle of the ethmoid plate.

Likewise, the prospective maxillary region at the 15 ss was labeled (Figure 5B) and traced chronologically by 30 ss (Movies S2 and S3). The labeled CNCC at 15 ss (Figure 5B) migrated along the ventrolateral side of the eye and destined to the maxillary region at 30 ss (Movies S2 [lateral view] and S3 [dorsal view]). To examine lineage of the maxillary region after 30 ss, the maxillary region at 24 hpf (30 ss) (Figure 5D magenta) in *sox10:Dendra2* was photoconverted, enabling us to trace its destination at 48 and 72 hpf. The result showed that this photoconverted region (magenta) was differentiated and incorporated into the lateral part of the ethmoid plate at 48 hpf (Figure 5D′), where it persisted at 72 hpf (Figure 5D″). This evidence shows that the majority of the prospective maxillary region comprises the lateral part of the ethmoid plate. Compared with the previous studies, we provided a chronological lineage tracing (time‐lapse imaging) of CNCC in both the frontonasal pathway and maxillary pathway from 15 to 30 ss. Therefore, the ethmoid plate consists of CNCCs derived from the frontonasal and maxillary regions.

### 2.5 *zfhx4* reduction results in defects in CNCC development

**FIGURE 6:**
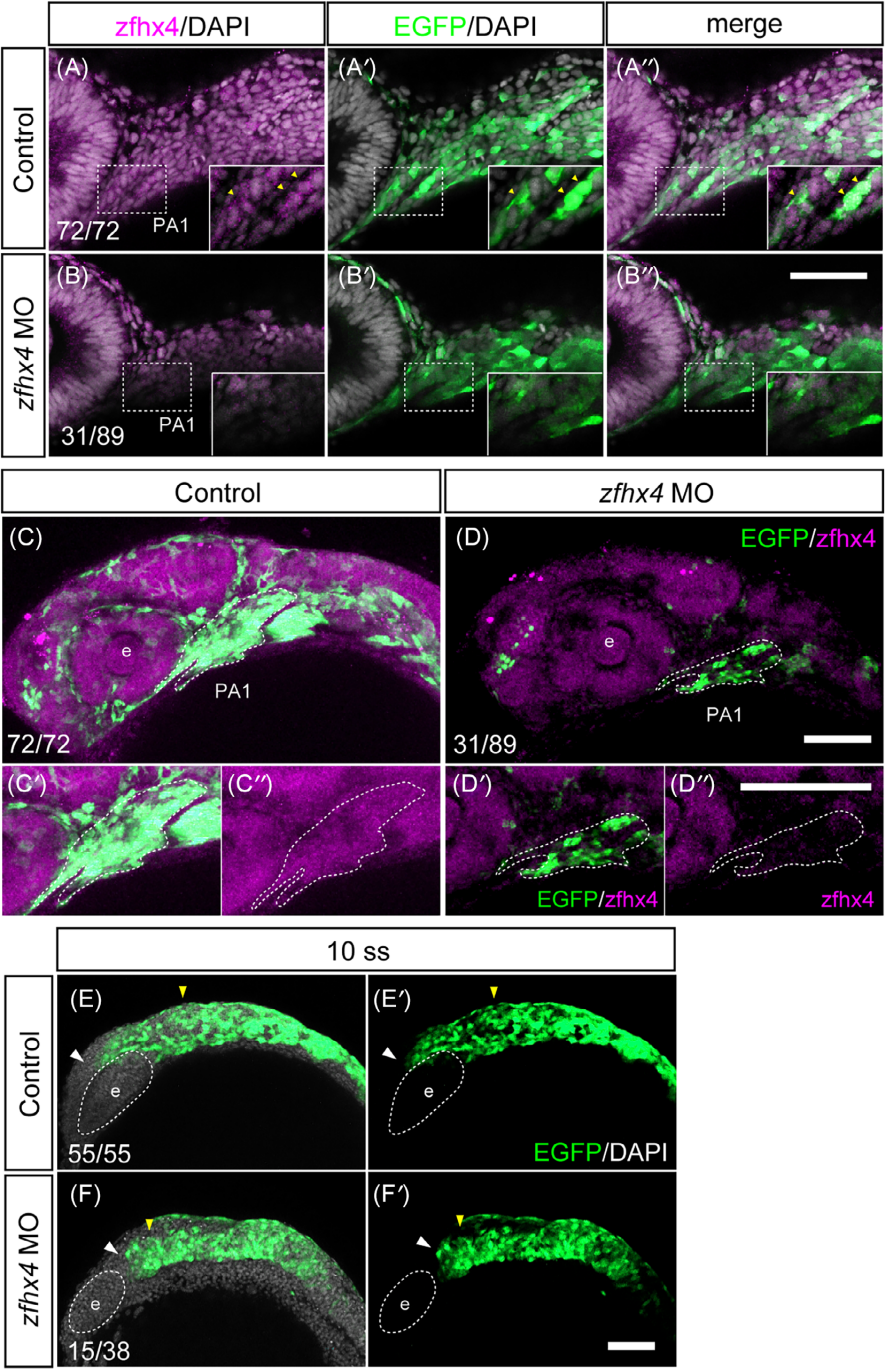
*zfhx4* morphants resulted in defective cranial neural crest cell (CNCC) development and pharyngeal arch formation. (A–D″) Immunofluorescence images of the control and *zfhx4* MO (E2I2)‐injected *sox10:EGFP*. Samples were stained with anti‐GFP antibody (NCCs), anti‐*zfhx4* antibody, and 4′,6‐diamidino‐2‐phenylindole (DAPI, nucleus) at the 10‐somite stage (ss) and 24 hpf. (A–A″) zhfx4 protein demonstrated nuclear localization in the NCCs. Insets show a magnified view of the first pharyngeal arch (PA1, white dotted lines). Yellow arrowhead shows the co‐localization of the CNCCs with the zfhx4 protein. (B–B″) *zfhx4* morphants showed decreased *zfhx4* expression in the CNCCs. (C,D) *zfhx4* morphants showed a decreased number of NCCs, leading to PA1 morphological defects. White dotted lines represent PA1 at 24 hpf. (C′–D″) Magnified view of PA1 in (C,D). (Control: *N* = 72, MO [E2I2]: *N* = 89). (E–F′) CNCC migration defect in *zfhx4* morphants. *zfhx4* MO was injected into *sox10:EGFP* embryos. Migrating CNCCs were visualized usin*g sox10:EGFP* at the 10 ss. The yellow arrowhead represents the position of the anteriormost midbrain and hindbrain, which are the starting sites for migrating CNCCs. The white arrowhead indicates the front edge of the migrating CNCCs. (Control: *N* = 55, MO [E2I2]: *N* = 38). e, eye; PA1, first pharyngeal arch. Scale bars: 100 μm in (C, D, E–F'); 50 μm in (A–B'', C'–D'').

We further investigated the detailed mechanism of craniofacial defects by reducing *zfhx4* from an earlier stage of zebrafish embryos, using the advantageous feature of imaging CNCCs in *sox10:EGFP* zebrafish embryos. In the control embryos, *zfhx4* was strongly expressed in the cranial neural crest population of PA1, where sox10‐EGFP exhibited a positive signal (Figure 6A–A''). In contrast, *zfhx4* morphants showed a substantially reduced *zfhx4* expression in the neural crest cells (Figure 6B–B''). PA1 was filled with sox10‐EGFP and *zfhx4* double‐positive cells in the control (Figure 6C–C''). The *zfhx4* morphants exhibited decreased *zfhx4*‐positive CNCCs, leading to substantial PA1 malformation, and subsequent abnormalities in the craniofacial skeleton, including the maxilla, palate, and mandible (Figure 6D–D''). Therefore, *zfhx4* possibly also plays a role in CNCC development. Thus, we investigated the migratory stage of CNCCs following MO treatment and observed an inhibitory effect on CNCC migration in the *zfhx4* morphants (Figure 6E–F'). These results indicated the significance of *zfhx4* in CNCC migration, and any impact on migration led to PA1 malformation. Therefore, the inhibitory effect and/or delay of CNCC migration leading to PA1 malformation, which affect facial primordia formation, underlie the etiology of craniofacial defects in zebrafish.

## 3 DISCUSSION

Recent advances in genomic sequencing technologies have unveiled the genetic underpinnings of several congenital diseases. Along with the demand for models for analyzing new mutations, this surge in information has driven the development of several cellular and animal models.^26^ However, multicellular models are preferred for craniofacial defects like orofacial clefts because of their structure and morphogenesis complexity. Thus, mice and zebrafish have been widely used for this purpose.^27^ The similarity of the craniofacial development process between these two species has been previously discussed.^3^, ^8^, ^28^ Particularly for the maxillary component, the ethmoid plate in zebrafish has been described as a homologous structure to the secondary palate in mammals, whereas the medial ethmoid plate corresponds to the primary palate with a similar cellular origin.^3^, ^9^, ^29^ However, the ethmoid plate in zebrafish is anatomically classified as the primary palate because the nasal cavity is not formed in fish; thus, the oral cavity is not separated from the nasal cavity by the secondary palate. This discussion remains open, and further investigation is essential.

We addressed this knowledge gap by comprehensively comparing gene expression profiles during craniofacial embryogenesis in mice and zebrafish. *Sox9* is a master regulatory transcription factor for chondrogenesis, marking ethmoid plate development in zebrafish.^17^ Therefore, we used *Sox9* as a molecular reference for palatogenesis and analyzed temporal trends to compare zebrafish and mouse embryonic development. Consequently, 86 genes with expression trends similar to those of *Sox9* in both species during embryonic development were selected using computational filtering. A similar temporal expression pattern during embryo development indicated that these genes exhibited similar biological functions.^30^ Gene regulatory network analysis using these selected molecules showed the GO terms, including “Embryonic skeletal system development” and “Mesenchyme cell proliferation,” which correlate highly to normal palatogenesis. After a systematic literature review, we refined the list to include five genes that (1) were strongly expressed during mice embryonic craniofacial development and (2) contained defects associated with signaling pathways involved in congenital craniofacial defects (*Sox9*, *Zfhx4*, *Zfhx3*, *Cjun*, and *Six1*). However, the precise nuances of their expression patterns and functional contributions to zebrafish craniofacial development remain elusive and warrant further analysis.

Notably, the genes that exhibited high expression during mouse secondary palate development, such as *Zfhx4* and *Cjun*, were also expressed in the ethmoid plate of zebrafish. Additionally, some genes expressed in the primary palate or nasal septum in mice, such as *Sox9*, *Zfhx4*, and *Six1*, were expressed in the medial ethmoid plate in zebrafish. Considering CNCC dynamics during the palate and ethmoid plate formation in both species are similar, which cellular source is derived from the frontonasal and maxillary prominence^8^, ^11^, ^13^, ^14^, ^15^, ^16^ (Figure 5). These results provide supplementary evidence to corroborate previous reports, which have shown that representative genes, such as *Tbx22*, *Osr1*, *Osr2*, and *Pax9*, in mice palate development also exhibit expression in developing maxillary component (“palate”) in zebrafish.^17^ However, some genes do not completely follow this rule and only show expression in one tissue, such as *Zfhx3* expression in the ethmoid plate in zebrafish but limited expression in the secondary palate in mice. In summary, we identified a new gene set whose expression pattern correlated with analogous tissues in mice and zebrafish during craniofacial development. These data suggest that the ethmoid plate (trabeculae) in zebrafish has molecular characteristics similar to that of the secondary palate in mice.

Another interesting aspect is how these genes are functionally conserved among species during palatogenesis. *Zfhx4* has been identified in mice and humans, and its mutations lead to orofacial clefts.^22^, ^23^ Therefore, we selected *Zfhx4* to assess the conserved function during craniofacial development in mice and zebrafish. Severe deformation of the palatine bone, a major skeletal component of the secondary palate in mice, was observed, whereas zebrafish exhibited severe truncation of the ethmoid plate. Several researchers have argued the conserved function of genes between mice and zebrafish by comparing the phenotypes resulting from corresponding gene mutations. For example, some genes with mutations that could lead to human orofacial clefts, such as *IRF6* and *WNT9A*, showed a consistent phenotype in the ethmoid plate, indicating a well‐conserved function in palatogenesis in mice and zebrafish.^8^, ^9^ The results of the present study also provide evidence for another key molecule involved in palatogenesis in humans, mice, and zebrafish. Conserved mechanisms for embryonic craniofacial development between these species are not limited to palatogenesis but also apply to jaw patterning via the endothelin signaling pathway by patterning the developing CNCCs.^4^, ^28^

Interestingly, *Zfhx4* knockout mice also exhibited a substantial reduction in osteogenesis in the mandibular body compared with the control, which may recapitulate the mandibular phenotype in *zfhx4* morphants of zebrafish.

Reduced *sox10*‐positive CNCCs were observed in the zebrafish pharyngeal arch treated with the *zfhx4* antisense MO. Cleft palate etiology in *Zfhx4* knockout mice is considered a defect in the growth of palatal shelves and a failure to remove the tongue from the center of developing palatal shelves that produce mechanical inhibition of palatal fusion.^22^ Our results indicate that the craniofacial anomalies observed in *Zfhx4*‐deficient individuals are also caused by cranial neural crest development defects, requiring further confirmation. These results indicate the significance of using different disease models for disease, considering their respective biological advantages, to reveal a wide spectrum of cellular mechanisms underlying the morphological defects of craniofacial diseases.

A limitation of this study is the use of different species in varying environments to directly compare molecular profiles and phenotype during embryonic craniofacial development. Gene expression profiles during embryonic development have diverged considerably throughout evolution, necessitating caution when comparing different species models. Additionally, *Sox9*, the gene selected as a reference to investigate the molecular network for palatogenesis in this study, is not exclusively expressed in the CNCCs and skeletal structure of craniofacial areas.

*Zfhx4* was the only gene functionally assessed in both species in this study. Therefore, further functional validation using alternative genes is necessary to elucidate the utility of these models in investigating orofacial cleft etiology in diverse contexts. Additionally, *osterix* zebrafish mutants exhibit specific defects in the anterior skull and upper jaw, and the top of the skull comprises a random mosaic of bones derived from individual initiation sites.^31^ Furthermore, *osterix* and *runx2* are co‐expressed in zebrafish, which suggests that *osterix* interacts with *runx2*, similar to the interaction of Osterix with Runx2 in mice.^22^, ^31^ Thus, a detailed analysis of the interaction between *zfhx4* and osteogenic factors such as *osterix* and *runx2* in zebrafish will be necessary for revealing the similarity of the osteogenic molecular network with that in mice in our future research.

In conclusion, we identified novel gene sets demonstrating similar expression patterns during embryonic craniofacial development in mice and zebrafish. Additionally, we showed the functional similarity of *Zfhx4* in the embryonic craniofacial development of mice and zebrafish, in which loss‐of‐function resulted in distinct craniofacial defects, such as cleft palates in mice and ethmoid plate deformation in the zebrafish. These results support the existing hypothesis of similarities in the craniofacial development between zebrafish and mice from a new perspective. Thus, *ZFHX4* may play conserved roles in the embryonic development of zebrafish and mouse maxilla and could reveal the disease etiology associated with *ZFHX4* mutation, demonstrating the usefulness of the two disease models in modeling craniofacial defects in humans.

## 4 EXPERIMENTAL PROCEDURES

### 4.1 Test species

Institute of Cancer Research (ICR) mouse embryos (CLEA, Tokyo, Japan) were used for in situ hybridization and immunohistochemical analyses. *Zfhx4* null and wild embryos were collected from pregnant *Zfhx4* heterozygous female mice.^22^ The study was reviewed and approved by the Committee on the Ethics of Animal Experiments of Osaka University Graduate School of Dentistry and the Ethics Committee of Osaka University. All mouse experiments were conducted in Osaka University.

The zebrafish (*Danio rerio*) RIKEN WT (RW) and *Tg* (*sox10:EGFP*) and *Tg* (*sox10:Dendra2*) (RW background; referred to as *sox10:EGFP and sox10:Dendra2*, *respectively*) strains were maintained under a 14‐h light/10‐h dark cycle. The water temperature was kept at 28 ± 1°C, and water quality conditions were maintained according to The Zebrafish Book (University of Oregon Press) and the Guide for the Care and Use of Laboratory Animals 8th edition (National Research Council, 2011). These sox10‐reporter lines were maintained as heterozygotes and homozygotes.^21^

### 4.2 Morpholinos

MO antisense oligonucleotides were designed to block splicing, resulting in the skipping of exon 2 (i.e., the exon 2 splice donor site designated as E2I2), and to block the translational initiation of *zfhx4* (Gene Tools, Philomath, OR, USA). The MO sequence was as follows: *zfhx4* (E212), 5′‐AAGGGAAAACTACTCACCATGACCA‐3′ and *zfhx4* (ATG) 5′‐GGATCTCATTTCATCCAGCCTGTCA‐3′. MOs were diluted to 1 μg/mL and injected into one‐cell‐stage zebrafish embryos. The morphological abnormalities were evaluated at 24 and 96 hpf.

### 4.3 Whole‐mount in situ hybridization

Whole‐mount in situ hybridization of mouse embryos was performed as previously described.^32^ The dissected embryos were fixed in 4% paraformaldehyde (PFA; Wako, Tokyo, Japan) at 4°C and stored in 100% methanol at −30°C. The primer sequences for the RNA riboprobes were selected from the Allen Brain Atlas (https://mouse.brain-map.org/). After hybridization with the digoxygenin (DIG)‐labeled riboprobe, BCIP/NBT was used to visualize the alkaline phosphatase‐conjugated anti‐DIG antibody (Roche, Basil, Switzerland). A minimum of three embryos were examined per probe.

For the zebrafish, RNA probes for in situ hybridization, including s*ox9b*, *zfhx4*, *zfhx3b*, *cjun*, and *six1b*, were synthesized according to the following protocols: Partial cDNA sequences of 1500 bases with T7 and T3 promoter sequences as templates for RNA probes were synthesized using integrated DNA technology. Antisense riboprobes were synthesized with a T7 MEGASCRIPT using DIG‐labeled UTP (Roche) according to the manufacturer's protocol.

Zebrafish embryos were fixed at 48 hpf for 2 h with 4% PFA (Wako) in 1× phosphate‐buffered saline (PBS; Invitrogen, Waltham, MA, USA) and dehydrated for >2 h in ice‐cold methanol (MeOH, Wako) at −20°C. The embryos were rehydrated stepwise with 80%, 50%, and 20% MeOH in PBS‐T containing 0.1% TritonX‐100 (Cayman Chemical, Ann Arbor, MI, USA) on ice and placed back in PBS‐T. The samples were processed to remove pigmentation by bleaching with 3% hydrogen peroxide (Wako) and 0.5% potassium hydroxide (Wako) under light for 2 h. After bleaching, the samples were incubated with 10 mg/mL of protease type XIV (Sigma‐Aldrich, St. Louis, MO, USA) in PBS‐T for 30 min. The samples were then postfixed in 4% PFA for 20 min. After postfixation, samples were washed with 150 mM Tris–HCl (pH 8.5) for 5 min, heated for 15 min at 70°C, and washed twice with PBS‐T for 5 min. Samples were then incubated in ice‐cold acetone (Wako) for 20 min at −20°C. The embryos were prehybridized for at least 1 h at 60°C in a hybridization buffer (50% formamide [Nacalai Tesque, Kyoto, Japan], 10% dextran sulfate [Sigma‐Aldrich], 5× saline‐sodium citrate [SSC] pH 7.0 [Nippon Gene, Tokyo, Japan], 10% sodium dodecyl sulfate [SDS; Wako], 50 mg/mL heparin [Sigma‐Aldrich], 50 mg/mL tRNA [Roche], and 0.1% Tween‐20). Hybridization was performed in the hybridization buffer containing 500 ng of probe overnight at 60°C. Samples were washed twice with wash buffer I (50% formamide, 2× SSC [pH. 4.5], 1% SDS, and 0.1% Tween 20) for 15 min at 60°C and then washed twice with wash buffer II containing 500 mM NaCl, 10 mM Tris–HCl, and 0.1% Tween for 15 min at 60°C. The samples were subsequently blocked with 2% goat serum (Gibco, Waltham, MA, USA) and 2 mg/mL bovine serum albumin (BSA; Wako) in PBS‐T for 2 h and incubated overnight at 4°C with the preabsorbed alkaline‐phosphatase‐coupled anti‐digoxigenin antiserum (Roche) at 1/1000 dilution in blocking buffer. Finally, the samples were washed six times with PBS‐T for 15 min. The detection solution (450 mg/mL NBT and 175 mg/mL BCIP; Roche) in the alkaline phosphatase reaction buffer (100 mM Tris–HCl [pH 9.5], 50 mM MgCl_2_, 100 mM NaCl, and 0.1% Tween 20) was used for detection. The samples were washed with PBS‐T thrice for 5 min and then the reaction was stopped. They were then incubated with 4% PFA for 30 min. All samples were transferred into 90% glycerol in PBS for observation and imaged on a Leica M80.

### 4.4 Fluorescence imaging and immunofluorescence staining

In mice, immunostaining of frozen sections was performed as previously reported.^33^ The following antibodies were used: rabbit polyclonal anti‐*SOX9* (Millipore Sigma, Burlington, MA, USA; AB5535, 1:500), rabbit polyclonal anti‐*ZFHX4* (Abcam, Cambridge, UK; ab254654, 1:400), rabbit polyclonal anti‐*SIX1* (Millipore Sigma; HPA001893, 1:500), rabbit monoclonal anti‐*CJUN* (Cell Signaling Technology, Danvers, MA, USA; 9165, 1:400), and Alexa 546‐labeled donkey anti‐rabbit IgG (Invitrogen; A10040, 1:500).

Immunofluorescence staining was performed as previously described (Narumi et al., 2020)^34^, with minor modifications. Zebrafish embryos were fixed with 4% PFA (Wako) at 10 ss, 24, and 96 hpf and treated with 100% ice‐cold methanol (Wako) at −20°C for longer storage. The fixed earlier‐stage embryos (< 96 hpf) were permeabilized with 1% TritonX‐100 in PBS for >1 h, and 96 hpf samples were prepared as previously described. After blocking with 3% BSA in PBS‐T (0.1% TritonX‐100 in PBS) for 2 h, the embryos were incubated with mouse anti‐GFP (1/1000, Invitrogen; AB_221568, 1/1000 Millipore; MAB3580), rabbit anti‐zfhx4 (1/500, Invitrogen; AB_2692007), and mouse anti‐collagen type II (anti‐coll2, 1/100, DSHB; AB_528165) primary antibody or PNA Alexa Fluor 488 conjugate direct labeling (1/1000, Thermo Fisher Scientific, Waltham, MA, USA) overnight at 4°C. The samples were washed six times with PBS‐T for 15 min and stained with the following secondary antibodies: Alexa Fluor 488‐goat anti‐mouse, Alexa Fluor 568‐goat anti‐rabbit (1/1000, Life Technologies, Waltham, MA, USA), and 4′,6‐diamidino‐2‐phenylindole solution (1/1000, DOJINDO, Kumamoto, Japan) overnight at 4°C. After washing six times with PBS‐T for 15 min, the samples were embedded in 1% low‐melting agarose and mounted on a 27‐mm noncoated glass‐bottom dish (IWAKI, Shizuoka, Japan). For time‐lapse imaging, the samples were anesthetized with 0.02% MS‐222 (Sigma‐Aldrich) and embedded in 1% low‐melting agarose containing 0.02% MS‐222 on the glass‐bottom dish.

All immunofluorescence images were acquired using a Zeiss LSM800 system with Zeiss ZEN black or blue software. All procedures were performed at room temperature unless otherwise specified.

### 4.5 RNA extraction

Each stage of ICR mice and RW zebrafish embryos was dissected, and the total RNA was extracted using an RNeasy kit (Qiagen, Hilden, Germany) according to the manufacturer's protocol. mRNA was extracted individually to conduct separate RNA‐Seq analyses at multiple developmental stages (E8.5, E9.5, E10.5, E11.5, E12.5, and E13.5 in mice, and 20 ss, 24, 32, 48, and 72 hpf in zebrafish). For each developmental stage, 11 individual mouse embryos were used except for E9.5 (29 individuals). In zebrafish, 16 pooled embryos (30 individuals/pool) were used except for 32 hpf (8 pooled embryos).

### 4.6 RNA‐Seq library preparation and sequencing

Lasy‐Seq version 1.1 protocol (https://sites.google.com/view/lasy-seq/) was used to perform 3′RNA‐Seq.^35^, ^36^ Briefly, 180 ng of total RNA was reverse‐transcribed using an RT primer with an index and SuperScript IV reverse transcriptase (Thermo Fisher Scientific). All RT mixtures were pooled and purified using an equal volume of AMpure XP beads (Beckman Coulter, Brea, CA, USA) according to the manufacturer's instructions. Second‐strand synthesis was conducted with the pooled samples using RNaseH (5 U/μL; Enzymatics, Beverly, MA, USA) and DNA polymerase I (10 U/μL; Enzymatics). The mixture was subjected to RNase treatment using RNase T1 (Thermo Fisher Scientific) to avoid the carryover of large amounts of rRNA. Subsequently, the samples were purified using 0.8× volume of AMpure XP beads. Fragmentation, end‐repair, and A‐tailing were performed using 5× of the WGS fragmentation mix (Enzymatics). The Adapter for Lasy‐Seq was ligated using a 5× ligation mix (Enzymatics), and the adapter‐ligated DNA was purified twice with a 0.8× volume of AMpure XP beads. After optimizing the PCR cycles for library amplification using qPCR with EvaGreen, 20× in water (Biotium, Fremont, CA, USA) and the QuantStudio5 Real‐Time PCR System (Applied Biosystems, Waltham, MA, USA), the library was amplified using KAPA HiFi HotStart ReadyMix (KAPA BIOSYSTEMS, Wilmington, MA, USA) on the ProFlex PCR System (Applied Biosystems). The amplified library was purified using an equal volume of AMPure XP beads. One microliter of the library was subjected to electrophoresis using a Bioanalyzer 2100 with the Agilent High Sensitivity DNA kit (Agilent Technologies, Santa Clara, CA, USA) to assess quality. Subsequently, 150‐bp paired‐end reads were sequenced using HiSeq X Ten (Illumina, San Diego, CA, USA).

### 4.7 Mapping and gene quantification

PRJNA725414 was downloaded from https://www.ncbi.nlm.nih.gov/ for the time‐course RNA‐Seq of mouse embryonic development. Read 1 reads were processed with fastp (version 0.21.0)^37^ using the following parameters: –trim_poly_x ‐w 20 –adapter_sequence = AGATCGGAAGAGCACCGTCTGAACTCCAGTCA –adapter_sequence_r2 = AGATCGGAAGAGCGTCGTGTAGGGAAAGAGTGT ‐l 31. The trimmed reads of mouse and zebrafish were subsequently mapped to Mus_musculus.GRCm38.cdna.all.fa and Danio_rerio.GRCz11.cdna.all.fa, respectively, and deposited at http://asia.ensembl.org/index.html, using BWA mem (version 0.7.17‐r1188)^38^ with default parameters. The read count for each gene was calculated with salmon using ‐l IU, which specifies the library type (version 0.12.0).^39^

### 4.8 Identifying orthologous genes with temporal expression dynamics similar to those of *Sox9*

Orthologous genes were defined based on the gene names in the Ensemble database. Pearson's correlation coefficients of temporal gene expression between orthologs and *Sox9* were calculated using R (version 4.0.1) and an R function “cor”^40^ based on a list of gene quantification of each mouse and zebrafish ortholog. Sixty‐eight orthologs had Pearson's correlation coefficients >0.7. GO enrichment analysis was conducted against the 86 mouse orthologs using clusterProfiler (version 4.2.2)^41^ and the org.Mm.eg.db (version 3.14).

### 4.9 Micro‐CT analysis

Mouse embryos were fixed with Bouin's fixative solution, a 15:5:1 mixture of water‐saturated picric acid and concentrated formalin and acetic acid. Samples were stored in 70% ethanol and soaked in 1% phosphotungstic acid/70% ethanol as the contrast agent. The samples were scanned using Scanxmate‐E090S (Comscantechno, Yokohama, Japan) and rotated 360° in increments of 0.3°, generating 1200 projection images of 992 × 992 pixels. The surfaces of the maxillary and palatine bones were reconstructed by manually tracing the micro‐CT data using ITK‐SNAP (National Library of Medicine and National Institutes of Health, Bethesda, MD, USA).

## CONFLICT OF INTEREST STATEMENT

S.L., R.N., Y.N., M.M., and J.T. are employed by Kao Corporation.

## Supporting information


**Movie S1.** Time‐lapse imaging of the prospective frontonasal prominence from 15 to 30 ss. The left side of the prospective frontonasal prominence was photoconverted. A lateral view of *sox10:Dendra2* was recorded.

**Movie S2.** Time‐lapse imaging of the prospective maxillary prominence from 15 to 30 ss. The left side of the prospective maxillary prominence was photoconverted. A lateral view of *sox10:Dendra*2 was recorded.

**Movie S3.** Time‐lapse imaging of the prospective maxillary prominence from 15 to 30 ss. Both side of the prospective maxillary prominence were photoconverted for clear imaging. A dorsal view of *sox10:Dendra2* was recorded.

**Table S1:** Identified orthologous genes with a Pearson's correlation coefficient >0.7 for gene expression levels between Sox9 in mice and sox9b in zebrafish.

**Table S2:** Significantly enriched gene ontology (GO) terms (FDR = 0.05) of the 86 genes showing high correlation with Sox9 in mice and sox9b in zebrafish.

## Data Availability

All RNA‐Seq data are deposited as GSE254071 in Gene Expression Omnibus (GEO).
